# What contextual features affect the outcome and sustainability of therapeutic patient education interventions?

**DOI:** 10.1371/journal.pone.0292360

**Published:** 2024-02-14

**Authors:** Anne-Christine Rat, Laetitia Minary, Carole Ayav, Joelle Kivits, Laetitia Ricci

**Affiliations:** 1 Inserm, COMETE, PFRS, Caen Normandie University, Caen, France; 2 Rheumatology Department, University Hospital Center Caen, Caen, France; 3 APEMAC, Université de Lorraine, Nancy, France; 4 CHRU-Nancy, INSERM, CIC, Epidémiologie Clinique, Université de Lorraine, Nancy, France; University of KwaZulu-Natal College of Health Sciences, SOUTH AFRICA

## Abstract

**Background:**

Therapeutic patient education interventions are influenced by contextual factors. Therefore, describing the context is crucial to understanding how it can affect therapeutic patient education interventions and contribute to outcomes. We aimed to identify the contextual features that may affect the outcome and sustainability of therapeutic patient education interventions from a healthcare professional perspective.

**Methods:**

Semi-structured individual interviews were conducted with healthcare professionals involved in 14 therapeutic patient education interventions covering different chronic conditions (e.g., kidney and cardiovascular diseases, chronic pain, diabetes, obesity). Interviews were recorded and fully transcribed. We followed a general inductive approach to identify themes from healthcare professionals’ discourse to properly capture their perception.

**Results:**

Saturation was achieved with 28 interviews with 20 nurses, 6 dieticians, one physiotherapist and one psychologist. The average therapeutic patient education experience was 7 years. Identified contextual features clustered in 5 main themes: 1) conditions for the development of the intervention (genesis of the program: Who and what prompted it?; supports; content development; legislative framework); 2) integration of the program (in the healthcare pathway or the environment, relationship with the institution or local environment); 3) teamwork cohesion, interaction and integration with the environment (exchanges, cohesion of the team); 4) sustainability of the program; and 5) patient and healthcare professional contextual factors.

**Conclusion:**

New insights into contextual features that may be involved in therapeutic patient education interventions are represented in a framework based on the Medical Research Council evaluation framework. These features need to be addressed in studies of therapeutic patient education interventions and could help healthcare professionals build more effective interventions within the context. However, describing a list of elements of the context is not enough; analyses should also focus on how the contextual elements might affect an intervention and how they interact.

## Background

Therapeutic patient education interventions (TPEIs) are now recommended for numerous chronic diseases. According to the World Health Organization, TPEIs should enable patients to acquire and maintain skills that allow them to optimally manage their lives with their disease. They are designed “to help patients and their families understand the disease and the treatment, cooperate with health care providers, live healthily, and maintain or improve their quality of life” [1]. At a minimum, such programs should improve both patient health knowledge and literacy [2, 3], but TPEIs can be a way to cope with change in self-identity and to plan, pace and prioritize [4].

Before an intervention is implemented, proof of efficacy is needed to use resources for interventions shown to bring about desired outcomes and that the benefits outweigh the harm [5].https://www.jphres.org/index.php/jphres/article/view/577/276 Unfortunately, the content and context of the implementation of TPEIs continue to be inadequately and insufficiently described [6–8], which limits the transferability of their results [9–11] and therefore their diffusion in a real world, non-experimental setting. This situation is due in part to the continued use of simplistic linear evaluative approaches, which have focused on TPEI efficacy without exploring mechanisms by which they produce change [12]. This focus on efficacy presupposes that the context can be standardized. New approaches in intervention research underline the uncontrollable nature of the context and call for an explicit explanation of how the context may affect the intervention and contribute to its results [13]. Therefore, the context must be characterized.

Some tools and recommendations may help researchers characterize the context (e.g., the Template for Intervention Description and Replication (TIDieR) tool, the Intervention TAXonomy (ITAX), the Mechanisms of Action in Group-based Interventions [MAGI] Frameworks and ASTAIRE [14–20]). However, these tools rarely ask for details on the context of the intervention (e.g., in the MAGI framework, context is simply specified as “wider socio-cultural, economic, environmental, community and organizational factors”) or are developed in fields far removed from the clinical environment, which thus presents organizational specificities, particularly in terms of institutional constraints.

The “ClassificatiON des Composantes des programmes d’Education Thérapeutique” (CONCErTO) project aims at identifying medical, educational, psycho-sociological, organizational, and environmental elements that constitute a TPEI and that can have an impact on its effectiveness and sustainability. The focus is on providing a tool to describe TPEI in research publications and participate in understanding their mechanisms of action, especially in characterizing interactions between intervention and contextual factors. Better insight may help healthcare professionals (HCPs) build an effective and generalizable intervention within the clinical context.

The present study participates in the CONCErTO project by identifying the contextual features that may affect the outcome, participation and sustainability of a TPEI from an HCP perspective.

## Methods

### Design of the study

This study used a qualitative approach by using semi-structured individual interviews with HCPs involved in TPEIs between April 2016 and May 2017. The COnsolidated criteria for REporting Qualitative research (COREQ) Checklist was used to report the study [21].

### Sample

TPEIs were chosen among those certified by the Regional Health Agency of Lorraine Region (northeastern France). We chose not to include TPEIs that focused on psychiatric disorders or that included children. Among the remaining interventions, 12 were selected to maximize variation sampling [22, 23]. We also added 2 programs outside the region to account for the possible differences in practices and perspectives: one in Paris and one in Grenoble (Auvergne-Rhone-Alpes Region).

We used the following criteria to select TPEIs:

- Diseases targeted by the intervention: digestive cancers (n = 2), cardiovascular diseases (n = 2), kidney failure (n = 2), rheumatic diseases (n = 1), chronic obstructive pulmonary disease (COPD) (n = 1), asthma (n = 1), chronic pain (n = 1), multiple sclerosis (n = 1), hepatitis (n = 1), diabetes/obesity (n = 1), multiple pathologies (n = 1).
- Setting: hospital interventions (n = 9), non-hospital interventions (n = 5), urban interventions (n = 10), rural interventions (n = 4).

We interviewed all HCPs present on the day the interviewer was at the different sites.

We asked all HCPs involved in the selected interventions who were present on the day of the appointment to participate in the study. All HCPs agreed to participate.

#### Data collection

After having obtained informed consent, we first invited HCPs to complete descriptive information regarding their profession, age and number of years of experience in TPEI. Researchers explained the aim of the study but not their personal goals. One experienced female health psychologist (LR, PhD) and one experienced female sociologist (JK, PhD) conducted face-to-face interviews with HCPs where the interventions took place. The interviewers were not involved in any TPEI as stakeholders, but JK coordinated a support unit of TPEIs. The interviewers did not know any of the participants before the study start.

Interviews lasted about 1 hour. They were audio-recorded and transcribed verbatim. Only the interviewer and the HCP were present during the interviews. The interviews were semi-structured and conducted until data saturation, that is, until no new idea emerged, then data were completed with 3 more interviews [24]. None of the interviews were repeated and transcripts were not returned to participants for comments. Field notes were not taken during or after the interviews. Authors had no access to information that could identify individual participants during or after data collection.

The interview guide (Table 1) was built during 3 meetings between a clinician/epidemiologist, also coordinator of a TPEI (ACR); the psychologist (LR); and the sociologist (JK).

**Table 1:** Content of the interview guide.

**Opening questions**
“How do you practice TPE? What do you think makes TPE successful, what works? what is important? What makes TPE less successful?”
**Probing questions for in-depth exploration**
Organization/structure of the TPE
Methods, facilitation, HCP-patient relationship, pedagogical tools used, customization
Role of the institution
Integration in the department/network, support
Role of training
Impact of patient characteristics
Impact of social factors
Integration of theoretical aspects in patient education
Other aspects not addressed during the time of interview

TPE, Therapeutic Patient Education; HCP, healthcare professional

The key research questions for discussion were to describe TPEI practices and identify organizational, pedagogical, psychosocial, medical or contextual elements that may affect the TPEI outcome, participation and sustainability.

### Data analysis

Transcripts were loaded in the NVIVO V11 software.

We followed a general inductive approach [25] to identify themes from the HCPs’ discourse to properly capture their perception by using grounded theory as an overall methodology orientation for the thematic analysis process [26]. In this approach, the researchers read the raw data to generate themes of analysis (without a priori expectations) A theme was defined as a topic sufficiently distinctive for the researcher to be recognized as providing important meaning regarding the research question [27].

All transcripts were first read to obtain a global impression and to identify preliminary codes [28]. Then, the health psychologist (LR) proposed a first draft of the codebook based on the analysis of the first 3 interviews. Next, on the basis of 2 other interviews, the codebook was refined collegially during a meeting with the sociologist (JK) and the clinician/epidemiologist (ACR). In 2018, a psychology student (master’s degree) was trained on the codebook. Six interviews were first encoded by LR and the psychology student. All coding disagreements were resolved by discussion. A document specifying the content of the analysis categories was created and revised progressively to stabilize the codebook. Then the student coded the remaining data. Illustrative quotes for each theme were selected from these data.

### Ethical considerations

The protocol was approved by the Nancy Hospital-University Ethical Review Committee (CRENHU) on December 10, 2015. All participants gave oral consent to participate, including the publication of anonymous quotations, and signed an information and non-opposition document. All experiments were performed in accordance with relevant named guidelines and regulations.

## Results

Saturation was achieved with 28 interviews. Table 2 shows that sampling was sufficiently diverse to collect a large diversity of perceptions concerning TPEIs. All interviewed HCPs were involved in the delivery stage of the intervention.

**Table 2:** Description of the sample.

Disease	No. of interventions	Hospital/non-hospital intervention	Urban/rural intervention	Specific HCP profession	Age group, years	Years of TPE experience	
Digestive cancers	2	Hospital	Urban	Nurse	21–30	6	#1, nurse, 21–30 years old, female
		Hospital	Urban	Dietician	21–30	0.3	#2, dietician, 21–30 years old, female
Cardiovascular diseases	2	Hospital	Urban	Nurse	51–60	18	#3, nurse, 51–60 years old, female
		Hospital	Rural	Nurse	51–60	12	#4, nurse, 51–60 years old, female
				Dietician	31–40	3	#5, dietician, 31–40 years old, female
Kidney failure	2	Non-hospital	Urban	Nurse	61–70	8	#6, nurse, 61–70 years old, female
				Nurse	61–70	8	#7, nurse, 61–70 years old, female
		Hospital	Urban	Nurse	41–50	8	#8, nurse, 41–50 years old, female
				Nurse	51–60	8	#9, nurse, 51–60 years old, female
				Dietician	51–60	10	#10, dietician, 51–60 years old, female
				Dietician	51–60	6	#11, nurse, dietician,51–60 years old, female
Rheumatic diseases	1	Hospital	Urban	Nurse	51–60	12	#12, nurse, 51–60 years old, male
COPD	1	Non-hospital	Rural	Nurse	31–40	6	#13, nurse, 31–40 years old, female
Asthma	1	Hospital	Urban	Nurse	31–40	2.5	#14, nurse, 31–40 years old, female
				Nurse	51–60	6	#15, nurse, 51–60 years old, female
Chronic pain	1	Hospital	Urban	Nurse	61–70	3	#16, nurse, 61–70 years old, female
				Nurse	21–30	3	#17, nurse, 21–30 years old, female
				Nurse	21–30	1	#18, nurse, 21–30 years old, female
				Nurse	21–30	2	#19, nurse, 21–30 years old, female
Multiple sclerosis	1	Non-hospital	Urban	Nurse	51–60	10	#20, nurse, 51–60 years old, female
Hepatitis	1	Hospital	Urban	Nurse	31–40	6	#11, nurse, 31–40 years old, female
Diabetes/obesity	1	Non-hospital	Rural	Nurse	51–60	6	#22, nurse, 51–60 years old, female
				Nurse	41–50	13	#23, nurse, 41–50 years old, female
				Dietician	21–30	6	#24, dietician, 21–30 years old, female
				Psychologist	51–60	10	#25, psychologist, 51–60 years old, female
Multiple pathologies	1	Non-hospital	Rural	Nurses	41–50	8	#26, nurse, 41–50 years old, female
				Dietician	41–50	8	#27, dietician, 41–50 years old, female
				Physiotherapist	41–50	8	#28, physiotherapist, 41–50 years old, male

HCP, healthcare professional; TPE, therapeutic patient education; COPD, chronic obstructive pulmonary disease

Among the 14 interventions of interest, 10 proposed group sessions, 3 individual sessions and one both.

Contextual features that may affect the outcome, participation and sustainability of the TPEI mentioned by HCPs clustered in 5 main themes (Table 3): 1) conditions for the development of the intervention (genesis of the program [Who and what prompted it?], supports, content development, legislative framework); 2) integration of the program (in the healthcare pathway or the environment, relationship with the institution or local environment); 3) teamwork cohesion and interaction with and integration with the environment (exchanges, cohesion of the team); 4) sustainability of the program; and 5) patient and HCP contextual factors.

**Table 3:** Contextual feature themes.

Main themes	Sub themes, sub-sub themes
**1. Conditions for the development of the intervention**	
	**1.1 Genesis of the intervention: who and what prompted the TPEI** 1.1.1 Caregiver 1.1.2 Institution 1.1.3 Need felt
	**1.2 Supports** 1.2.1 Institutional support 1.2.2 Hierarchy 1.2.3 Colleagues 1.2.4 Training
	**1.3 Content development** Experiential knowledge and participatory approach
	**1.4 Legislative framework**
**2. Integration of the intervention in the healthcare pathway and the environment**	
	**2.1 Integration in the healthcare pathway** 2.1.1 Integration to the department in the hospital or in the regional healthcare network 2.1.2 Bridges between different specialties 2.1.3 Referral to community nurses and other HCPs 2.1.4 Links with referring physicians 2.1.5 Local integration and HCP network of the intervention**2.2 Integration in the environment** (department, institution or local environment): 2.2.1 Clinical department organization 2.2.2 Relationships/interactions with colleagues 2.2.3 TPEI is not a priority in regard to care 2.2.4 Lack of recognition, 2.2.5 Position and role of the health executives 2.2.6 TPEI activity report 2.2.7 Integration in the local environment
**3. Teamwork cohesion and interaction with and integration in the environment**	
	Cohesion of the team
	Organization
**4. Sustainability of the intervention**	
**5. Patient and HCP contextual factors**	

TPEI, Therapeutic Patient Education intervention; HCP, healthcare professional

### 1. Conditions for the development of the intervention

Who is behind the development of a TPEI, what is behind it, and who will support it will have an impact on the quality of care and the sustainability of the intervention.

#### 1.1 Genesis of the intervention: Who or What prompted the TPEI?

*1*.*1*.*1 Caregiver*. Most interventions were initiated by 1 or 2 caregivers, becoming the driving force of the intervention and generating momentum and commitment. The motivation and enthusiasm of these HCPs behind the TPEI is a positive factor for the development and quality of the projects.

*1*.*1*.*2 Institution*. Alternatively, the TPEI could be an initiative of the institution or the hierarchy. In this case, the provision of staff, equipment or various aids will be more assured. The institution’s policy can also more easily develop interactions between expert TPEIs, training, evaluation and quality policies to guarantee the constant improvement of programs: *“A caregiver’s position has been created to implement the Patient Therapeutic Education (TPE) intervention*.” [#1, nurse, 21–30 years old, female].

One intervention was solicited by regional public health authorities or the “agricultural social insurance”.

*1*.*1*.*3 Need felt*. One intervention targeting patients with COPD was developed from a field problem: *“the need was felt*.*”* [#13, nurse, 31–40 years old, female].

#### 1.2 Supports

*1*.*2*.*1 Institutional support*. Institutional support was highlighted. “Assistance has been provided by the person in charge of [the] TPEI in the healthcare institution. He helped us to organize our ideas, to prioritize, to list what we needed and to develop our tools.” [#11, nurse, 31–40 years old, female]. When the institution is the initiator of the project, its support is essential to ensure that it runs smoothly and continues to improve.

*1*.*2*.*2 Hierarchy*. The role of hierarchy was addressed; in one intervention the head of the department was a facilitator of the initiative of a nurse: *“The boss had understood that I was explaining little things to patients*, *little by little he was unloading some things to me and then I decided to do it for several patients and the intervention was set up like that*.” [#12, nurse, 51–60 years old, male]. The hierarchy can help with the organization and running of the project and can ensure interaction with the institution to facilitate the running of the project.

*1*.*2*.*3 Colleagues*. Colleagues in the clinical departments or those that met during training courses were a source of help: *“Colleagues came to the sessions to see how it was going…We have been able to exchange and each one has been able to bring ideas*.*”* [#11, nurse, 31–40 years old, female].

*1.2.4 Training*. **1.2.4.1 Support for the improvement or development of the intervention**. HCPs found that training courses and especially dissertations were useful to develop and implement their intervention. Training courses can be an opportunity to think about, refine and adjust interventions and tools: *“I passed the diploma*, *so that allowed me to better structure things and to come out of it with an established intervention*, *following requirements specification*.*”* [#12, nurse, 51–60 years old, male].

**1.2.4.2 Opportunity to discuss experiences and share supports**. Training courses are also an opportunity to meet colleagues, benefit from other experiences and share supports: *“Thanks to my colleagues who shared tools and standard templates with me during training courses*.*”* [#20, nurse, 51–60 years old, female].

When describing the contribution of training courses, HCPs highlighted their importance in refining and implementing their intervention, to adapt their attitude and behavior (e.g., facilitating a group, asking open-ended questions, getting patients to express themselves, having an open active listening posture) but not really to deepen their thinking or knowledge of theoretical models, pedagogical techniques or behavioral or therapies.

#### 1.3 Content development

*Experiential knowledge and participatory approach*. Intervention content was defined during meetings with different experts, HCPs and patients: *“We organized meetings with patient associations and physicians to identify aspects to be addressed in the intervention*.*”* [#2, dietician, 21–30 years old, female].

The content could be based on other interventions developed for other diseases.

Sometimes, TPEIs existed for many years but were not really structured and consistent. *“We have done TPE for a long time but without naming this activity*.*”* [#20, nurse, 51–60 years old, female].

#### 1.4 Legislative framework

Since 2010 in France, TPEIs have been regulated by a law, and the legislative changes have deeply modified the activity. Formal training of HCPs for a minimum of 40 hours is now mandatory. Interventions can be authorized only if they complete specified criteria of quality. These changes forced stakeholders to rethink what they were doing and to structure their intervention by documenting it: *“Before the program was authorized in 2012*, *I followed the patients*. *But it was less formalized*, *I didn’t have a chart*, *it was less rigorous*. *…The tools were already in use but have been adapted to make them more scientific*.*”* [#20, nurse, 51–60 years old, female]. Therefore, the legislative framework for interventions is important to improve TPEIs and ensure quality.

### 2 Integration of the intervention in the healthcare pathway and environment

#### 2.1 Integration in the healthcare pathway

For HCPs, integration of TPEI in the healthcare pathway was of major importance.

*2*.*1*.*1 Integration of the TPEI in the hospital department (or regional healthcare network)*. In the hospital, TPEIs can be part of usual care. Nurses on the ward are also TPEI nurses, TPEI sessions are organized at the same time as medical consultations or exams, and the physician on duty can be called in if necessary after a TPEI session. The information provided by the TPEI allows for knowing the patient better and is therefore useful to all the HCPs who care for the patient: *“In the hospital*, *TPEI is integrated into the medical care*: *consultation nurses are also TPEI nurses*, *TPEI sessions are organized at the same time as medical consultations*.*”* [#15, nurse, 51–60 years old, female]. *“The nurses on the ward take over in the evening if there is a need to explain again*.*”* [#1, nurse, 21–30 years old, female]. *“The doctor on duty is called in if necessary*, *to change the treatment*.*” [#4*, *nurse*, *51–60 years old*, *female]*. *“It’s once the intervention has started that the patient can begin treatment*.*”* [#11, nurse, 31–40 years old, female]. *“Often in the educational diagnosis*, *we have data that they don’t have and so we share with the nurses on the ward and that’s important too*. *If they have 5 entries*, *they will spend 15 minutes on each patient*, *whereas we will try to spend 45 to 60 minutes on the educational diagnosis to understand the patient*. *And this information is still useful information even for the nurses in hospital*.*”* [#1, nurse, 21–30 years old, female].

*2*.*1*.*2 Bridges between different specialties*. Bridges between different TPEIs have been implemented to improve the management of comorbidities: *“Patients already followed for diabetes can be included in a kidney TPEI because of the development of renal failure*.*”* [#6, nurse, 61–70 years old, female]. *“Patients can be referred to other*, *more appropriate TPE interventions to complement the intervention*.” [#7, nurse, 61–70 years old, female].

*2*.*1*.*3 Referral to community nurses and local HCPs not involved in the TPEI*. After the end of the TPEI, follow-up can be performed by a community nurse: *“A relay with a community nurse can be organized*.*”* [#1, nurse, 21–30 years old, female]. *“Patients can be referred to other professionals associated with the intervention or intervening in the medical department or being in the intervention’s network*: *e*.*g*., *psychologist*, *social worker*, *dietician*.*”* [#9, nurse, 51–60 years old, female].

Similarly, referral to other HCPs can be organized: *“Patients can be referred to other professionals associated with the program or intervening in the department to which the program is attached or by being in the program’s network*.” [#9, nurse, 51–60 years old, female].

*2*.*1*.*4 Links with referring physicians*. Links with attending physicians was frequently mentioned and was clearly associated with better results: *“If the doctor supports [the] TPEI and is involved*, *it works better*.” [#4, nurse, 51–60 years old, female].

*2*.*1*.*5 Local integration and HCP networks of the intervention*. Local integration and healthcare networks of the HCP of the intervention allow for recruiting patients and creating original initiatives as close as possible to patients’ daily life. Some interventions located in a rural area also try to recruit patients directly. However, they are still managed by HCPs working in networks and are still integrated in the healthcare pathway.

Overall, according to HCPs, integration of the intervention in the healthcare pathway has many advantages: *“Good local integration of the intervention*: *former fellows or acquaintances send patients*.*”* [#26, nurse, 41–50 years old, female]. *“Patients who have heard about the intervention through various communications*.*”* [#2, dietician, 21–30 years old, female]. *“A caregiver went so far as to say that it would be interesting to include the pharmacist so that the pharmacist could let the patients taste the dietary supplements and also thinks that it would be interesting for the nurses to be able to go to the patients’ homes*.*”* [#1, nurse, 21–30 years old, female]. *“The intervention will be presented to the inter-municipality to broaden the sources of recruitment with patients who would come from themselves*.*”* [#13, nurse, 31–40 years old, female].

#### 2.2 Integration in the environment (department, institution or local environment)

*2*.*2*.*1 Clinical department organization*. HCPs agreed that the TPEI should not disturb the functioning of the department. To be effective, TPEI should even be part of the ward’s care. HCPs work together in different roles, helping each other: *“[The] TPEI is organized to disrupt as little as possible the functioning of the department*, *which contributes to team cohesion and does not prevent a nurse from becoming seconded to do TPE*. Nurses on the ward do TPEI on the ward but *"we don’t force them*, *if they don’t have the time; we explain to them that it’s okay*, *and the reverse is also true*." [#1, nurse, 21–30 years old, female].

However, in practice this is not always easy.

*2*.*2*.*2 TPEI is not a priority in regard to care*. In the field, the TPEI is not a priority. In case of shortage of nurses, nurses will provide care at the expense of a TPEI, which will obviously be detrimental to the continuity of the intervention: *“For example*, *we were banned from TPEI for 3 weeks because we had to help other services [to replace colleagues on leave]*. *We have seen the difference between patients with or without TPEI*.*”* [#16, nurse, 61–70 years old, female].

The well-being of caregivers at work, in their relations with their colleagues and with their superiors, is also a guarantee of the program’s continuation and, perhaps also for the results.

*2*.*2*.*3 Relationships/interactions with colleagues*. Although information provided by the TPEI is shared with nurses on the ward or other HCPs constructively, a few HCPs described tensions with colleagues of the department but usually only when the intervention was being set up and for hospital-based TPEIs. Indeed, TPEI nurses were perceived as privileged and induced jealousy mainly because they had more time dedicated to the patients and were more autonomous: *"The start-up of TPEI was difficult in relation to colleagues since TPE nurses were seen as ‘privileged’; “Tensions are always felt when it comes to respect for each other’s attributions*." [#4, nurse, 51–60 years old, female].

*2*.*2*.*4 Lack of recognition*. Relations with colleagues are not always easy. A few HCPs involved in TPEIs felt a lack of recognition because their activity was not valued: "*[The] TPE is barely accepted*, *it earns 0 cents*… *in the establishment we are considered lazy because the TPE is blah blah blah blah*, *we just discuss*.” [#16, nurse, 61–70 years old, female].

*2*.*2*.*5 Position and role of health executives*. Healthcare executives have a number of important roles to play in supporting the development, implementation and operation of the TPEI. They must maintain the cohesion of the care team, support the project and the intervention, and be a promoter or leader of the intervention. HCPs particularly emphasized the role of healthcare executives, who must devote time to the TPEI or promote the activity. An unsupportive health executive or frequent turnover can jeopardize the life of a TPEI: *“The retirement of the health executive and the ensuing management turnover also failed to strengthen the position of [the] TPEI*.*”* [#19, nurse, 21–30 years old, female].

*2*.*2*.*6 Administrative tasks*, *documentation of TPEI activity*. The TPEI activity report was considered necessary to devote specific time for the TPEI. Among the regulatory requirements for the institution, regular reports must be filed to continue operating. However, these tasks are often considered time-consuming and performed at the expense of patient time: *“Making available dedicated time for TPEI is not always easy; it is thus important to document HCP activity to allow the health executive to make the case to management*.*”* [#12, nurse, 51–60 years old, male]. *“The problem is the analysis of this questionnaire [program evaluation]*, *the time to enter the data*. *I’m the only nurse here*. *So I spend a lot of time analyzing these questionnaires*. *It’s time you don’t have to spend on*.” [#20, nurse, 51–60 years old, female].

*2*.*2*.*7 Integration in the local environment*. Integration of the intervention in the local environment allows patients to go to the nearby gym or pool as part of the program and to continue the activity after the intervention: “*Links with local association allow patients for participating to the nearby gymnastic courses or going to the swimming pool;*, *“We should be sure that sport will be continued*. *Sport practice should be sustainable*.*”* [#28, physiotherapist, 41–50 years old, male].

### 3. Teamwork cohesion and interaction with and integration in the environment

Support was perceived primarily from within the TPEI team with the help of the hierarchy: *“Feeling part of a multi-professional structure (*…*) in a solid network*, *always having someone to refer to*, *even if they ask a question that’s not in my field*, *I can get information*, *and people really appreciate that*.*”* [#22, nurse, 51–60 years old, female].

Cohesion was strengthened by the TPEI organization. Exchanges could be formal during meetings or organized with liaison documents: *"An annual or biannual meeting is held with the coordinating physician of the program to improve it*.*"* [#13, nurse, 31–40 years old, female]. *"A liaison sheet is documented by the various program stakeholders at the end of the sessions*." [#22, nurse, 51–60 years old, female].

However, for interventions involving HCPs working in private practice, meetings were difficult to organize.

Patient exchanges can also be informal. However, formal exchanges are an opportunity to get to know the other parties involved in the intervention, which can increase interactions or referrals outside the intervention. In this way, new cooperative ventures and new care pathways can be created.

*"These moments are an opportunity to get to know each other and*, *consequently*, *to refer patients to other professionals working in different locations*.” [#1, nurse, 21–30 years old, female].

Regardless, HCPs agreed that informal exchanges should not be the only type of exchange: *“The informal dimension of exchanges when it is not accompanied by structured*, *organized exchanges makes exchanges unsystematic*, *which can be problematic*.*”* [#14, nurse, 31–40 years old, female].

Working in pairs can also strengthen the cohesion of the team.

### 4 Sustainability of the intervention

We identified 10 contextual elements of TPEI that could jeopardize sustainability.

#### 4.1 Non-authorization of the intervention by the regional health agency

Despite other ways of supporting patients and teaching them skills, when a TPEI is not authorized, HCPs will have no time to devote to this activity, and development of the project will be limited. The group dynamic will fade away because the TPE activity will not be structured and identified as different from care: *“Being authorized by the regional health agency brings something to the institution*.*”* [#16, nurse, 61–70 years old, female].

#### 4.2 TPE not a priority in the hospital’s strategy

When TPE is a priority in the hospital’s strategy, professionals can be assigned to support HCP teams in developing their program and in administrative tasks. Time will be devoted to this activity, training will be paid for by the institution, and exchanges will be organized. When TPE is not a hospital priority, the intervention relies on one or a few people, and risks disappearing in their absence or when they leave: *“Sustainability depends on the hospital’s strategy*.*”*[#8, nurse, 41–50 years old, female].

#### 4.3 Not a priority in regard to care

When HCPs are understaffed, care always comes before TPE: *“During the summer vacations*, *nurses had to intervene in other departments or replace colleagues who were absent in other departments*.*”* [#16, nurse, 61–70 years old, female].

#### 4.4 TPEI dependent on a driving HCP

Coordination and leadership of the TPEI must be shared and not rely on a single person. The development of a TPEI frequently relies on a driving HCP whose leadership role is to maintain team cohesion, promote innovation, organize activities and communicate. When the other HCPs of the team do not share these roles, for whatever reason, or when no one is designated to take over in case of absence, the TPEI stops: *“During the doctor’s sick leave*, *TPEI was not maintained*.*” [*#17, nurse, 21–30 years old, female]. *"TPEI is automatically included in the hospitalization for one of the doctors*. *The 2 other doctors have a different posture*, *TPEI is proposed to their patients but rarely accepted*.*"* [#19, nurse, 21–30 years old, female].

#### 4.5 Low involvement of the health executives

The specific role of healthcare executives is to advocate for TPE activity with the management and to obtain dedicated TPE working time. They can also take on the role of coordination and leadership and help with administrative tasks, but the latter two roles can be taken on by other HCPs: *“The administrative tasks ended up being the responsibility of the primary HCP*.*”* [#27, dietician, 41–50 years old, female].

#### 4.6 High turnover in teams

High team turnover does not strengthen team cohesion or long-term projects. Experience is also important for leading patient groups, for understanding patients’ difficulties and resources, and for appropriating teaching materials and techniques. The risk is that HCPs will become less involved, less motivated and more like executors: *“The retirement of the health executives and the subsequent turnover in leadership also did not strengthen the position of the TPEI*.*”* [#19, nurse, 21–30 years old, female].

#### 4.7 No specific time for TPEI

When no time is devoted to TPE, TPE can be part of working time but not a priority over care activities. TPE can also be done in addition to working time. Neither situation is viable in the long term: *“On Friday afternoons we stay in addition to our work time*.*”* [#15, nurse, 51–60 years old, female].

#### 4.8 Lack of documentation of TPE activity

Documentation of TPE activity is necessary to show what is being done, to show the patient involvement and to evaluate the intervention. These data help to show that the intervention is useful and worth pursuing and also to make the case for the TPE activity to management and to obtain dedicated TPE time: *“Making available dedicated time for TPE is not always easy*. *It is thus important to document HCP activity to allow the health executive to make the case to management*.*”* [#12, nurse, 51–60 years old, male].

#### 4.9 Absence of satisfaction or perceived effectiveness of TPEI by patients

Positive patient feedback on the TPEI is a reason to maintain the intervention: “*Patient satisfaction with their care is important*.” [#9, nurse, 51–60 years old, female].

#### 4.10 Therapeutic advances making the intervention useless

This case is rare. One example is the arrival of new treatments of hepatitis C that cure the infection, which is no longer a chronic disease: *“The arrival of new treatments in hepatitis C signals the end of the program in the short to medium term*.*”* [#14, nurse, 31–40 years old, female].

### 5 Patient and HCP contextual factors

Characteristics of patients (understanding and education, personality, motivation, social environment, beliefs and representation) and HCPs (medical knowledge, relational skills, pedagogical skills, training) that are also contextual factors or linked to contextual factors are developed elsewhere [29].

## Discussion

The contextual features that affected the outcome and sustainability of TPEIs clustered in 5 main themes: 1) conditions for the development of the intervention (genesis of the program [Who and what prompted it?], supports, content development, legislative framework); 2) integration of the program (in the healthcare pathway or the environment, relationship with the institution or local environment); 3) teamwork cohesion, interaction and integration with the environment (exchanges, cohesion of the team); 4) sustainability of the program; and 5) patient and HCP contextual factors.

**Fig 1:**
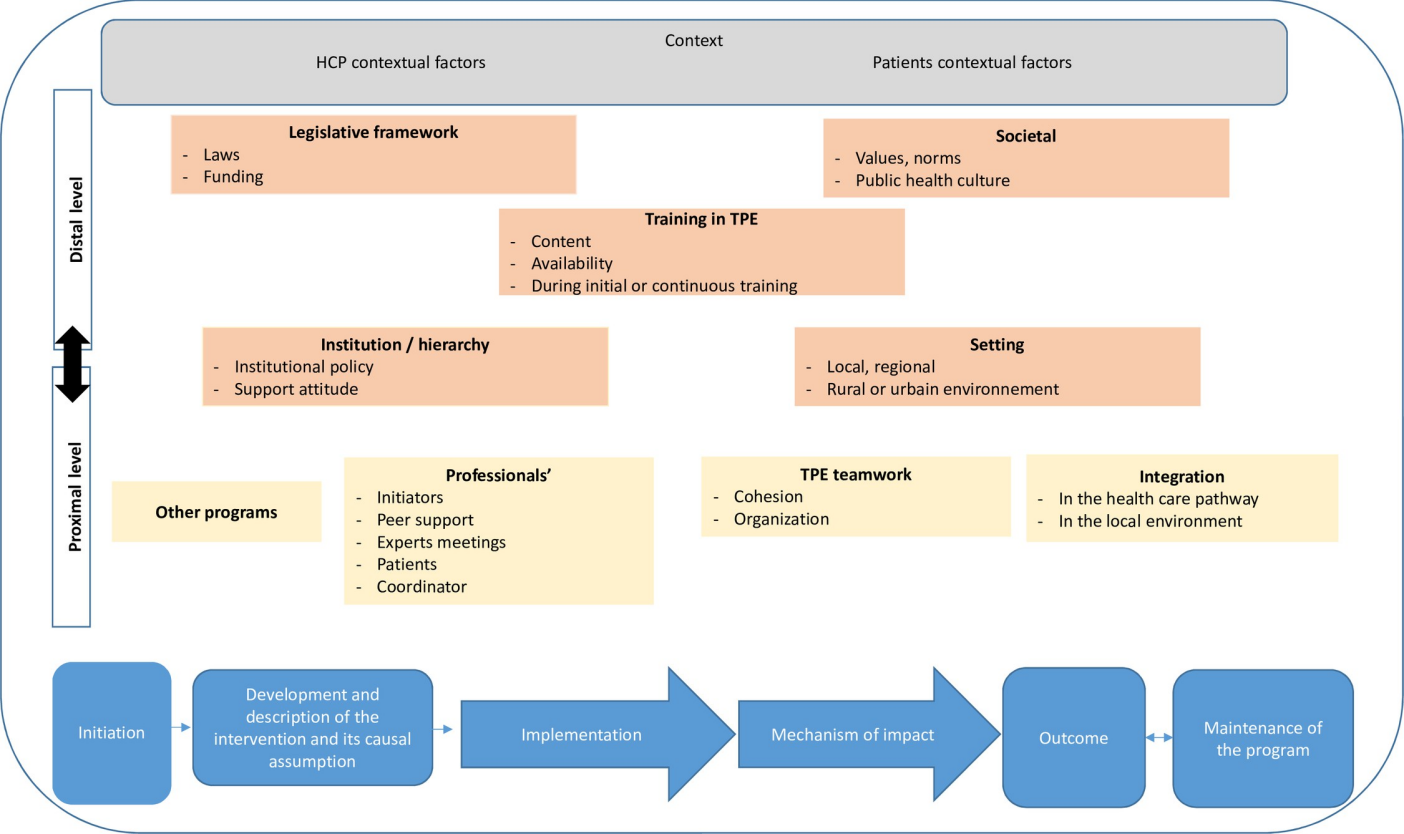
Representation of therapeutic patient education interventions in context. Blue boxes represent components of the process evaluation of the Medical Research Council evaluation framework to which we have added initiation and maintenance of the intervention. Yellow boxes are proximal contextual factors, and orange and then grey boxes more distal contextual factors. (Interactions between the different boxes are too numerous to be represented in a figure). HCP, healthcare professional.

From the findings of this study, we present new insights into the development of a framework following the Medical Research Council (MRC) evaluation framework [13] and describing elements of the context that can affect the life of a TPEI: its initiation, development, implementation, mechanism of impact, outcome and maintenance (Fig 1).

We have placed the various contextual elements that can affect the life of a TPEI on the MRC evaluation framework, ranking them from proximal (HCP, teamwork, other programs and integration) to distal (legislative and societal). We chose the MRC evaluation framework because it describes the process evaluation of complex interventions such as TPEI. It is the model best suited to the aim of our study, which is to identify the contextual characteristics likely to affect the outcomes and sustainability of a TPEI, to better describe the context of the intervention in the research publications and help HCPs implement more effective interventions in their context of implementation.

Process evaluations aim to provide a more detailed understanding that is needed to inform policy and practice. In MRC framework evaluation should address descriptions of initiation and development of the intervention, its implementation, mechanism of impact, outcome and context. The context is the external factors that can influence the delivery and functioning of interventions.

### Initiation

The impact of the context starts as soon as the initiation of the project. The institution can request a stakeholder to develop a project, but for most interventions, one driving HCP is at the origin of the project. Actually, the start of a TPEI is affected by HCP characteristics, societal features such as changes of patient–HCP relationships or patient empowerment and norms and values, legislative framework, institutions, integration of the TPEI in HCP training, development of TPEIs in numerous diseases, organization of the support to the TPEI and integration of the TPEI in the health care pathway.

### Development/implementation

The impact of regulations imposed by health authorities [30] improves and warrants the quality of the TPEI even if it is often perceived as a burden and disconnected from practical reality [31], as was also emphasized in our interviews. In France, a law has regulated the practice of TPEI since 2009. Any intervention must comply with a clear national specification, thus ensuring its relevance and quality [30].

The source of content development such as literature reviews or the need for assessment studies or theoretical models was rarely mentioned by the interviewed HCPs, although clinical practice guidelines state that interventions should have a theoretical framework and be evidence-based [32]. Most evidence-based approaches consisted of using a theoretical model for the whole intervention or for the therapeutic components used, by using a model to plan the whole development (e.g., the PRECEDE-PROCEED model [33]) or by using an intervention mapping approach [34]. Transferring an intervention implemented to another context was common, but the use of a structured process [10] of transfer seemed under-used.

The setting of the interventions [35] is important to consider during the development phase of the TPEI because the targeted population and environment will condition its content and form. Moreover, the setting strongly affects the politics and attitude of the organization or hierarchy toward the TPEI.

Relationships with the institution or department may sometimes be disappointing and may shape the intervention: motivation of TPEI stakeholders is not always shared by the other HCPs, and lack of recognition of their work and tensions with colleagues persist even if they usually improve over time. The integration of the TPEI in the initial training of HCPs could improve these tensions. A difficulty mentioned on a recurring basis in our study and in the literature was the lack of time because of competing responsibilities [31]. Health executives should be facilitators, allowing for the sessions to be held during work hours or obtaining dedicated HCP time for the TPEI. This was also highlighted in the Lemon et al. study [36].

The role of training is an interesting one. It provides a forum for exchanging ideas, thus allowing for distancing oneself from a program and enriching it.

HCPs highlighted the opportunity to share materials and adjust their interventions and tools. The dissertation required for the diploma is often an opportunity to develop or improve their TPEI. HCPs actually addressed practical contributions of the training in designing an intervention or implementing their activity (e.g., training in an appropriate relationship with patients, active listening, benevolence, use of open-ended questions). However, they did not address the training as a way to deepen their thinking or knowledge of theoretical models, pedagogical approaches or behavioral therapies, probably because theoretical understanding is less important to them than their practice, regardless, this is also a weakness of the training courses, which calls their content into question [37]. Smit et al., in their analysis of the development of primary-care interventions, highlighted the need to pay attention to the in-depth analysis of context, including the implementation of thorough and effective training for providers [38]. To describe TPEIs, details on the training seems important.

In our interviews, HCPs developed their TPEIs from existing knowledge and expertise from others. They indeed described the content development mainly as a replication of other interventions and meetings with experts. Lemon et al. also acknowledge the importance of asking for help from professionals with skills to develop TPEIs [36]. The importance of the role of patients in the intervention design and facilitation was highlighted in recommendations on TPE practice [30].

For HCPs, integrating TPEIs in the healthcare pathway is a contextual factor of major importance for different reasons. TPEIs can be proposed at the most appropriate time, they improve interactions among HCPs, and they allow for faster healthcare. Interactions between the city and hospital were little discussed; only referrals to community HCPs or links with referring physicians were mentioned. The current supply of TPEIs is still primarily hospital-based in France, although the authorities recommend new forms of cooperation based on the proximity principle [39]. According to Bresson, how city–hospital relations operate is not always highly structured, and the lack of a normative model of organization capable of responding to the different needs was considered an important explanatory reason for the lack of transversal organization of the educational process [40]. Of note, integration of TPE in the healthcare pathway is an evolving process. This integration in the healthcare pathway is dynamic with the multiple interactions between TPEIs and patients, providers, healthcare facilities, local environments, different networks and societal values and the constant adaptations.

### Mechanism of impact and outcome

All the different entities of the context discussed above will affect TPEI results directly or when interacting with the content and implementation of the TPEI. For example, in the Lelorain et al. study, HCPs regretted that TPE was not funded as for usual care, so it appeared less valuable and not part of medical care, which hinders its success [31]. Inter-professional cooperation as advocated in TPEIs [30] has rarely been evaluated even if modalities of communication of HCPs may have a role in the outcome. Finally, societal contextual factors were not addressed, although, patients’ perceptions of context, people’s social connectedness to others or trust toward the institutions and social norms affect health behavior and outcomes of a TPEI [41]. HCPs’ motivation also depends on the societal context and could be associated with the result [31, 36].

### Sustainability of the intervention

The role of a motor HCP, a key actor, has been particularly highlighted. Although this person is essential to start a project, an intervention can unfortunately become dependent on one person and its functioning and sustainability jeopardized. As expected, the role of the support of the institution and hierarchy and stakeholders were mentioned.

Finally, the societal context affects decision-makers and how they perceive the interest and value of an interventions [41].

Although these different contextual factors should be discussed to better describe TPEIs in research publications and to determine the mechanisms of action, descriptions of their interactions with the TPEI are even more important. Indeed, a TPEI can be understood as being formed from contextual entities and intervention entities that make the whole intervention and, in isolation or combination, can produce the outcome of the intervention [42, 43]. Intervention effects might result from interactions with the context, multiplication rather than addition processes and non-linear relationships [44]. The context of the intervention can itself be viewed as consisting of nested systems (organizational, social, cultural and geographical) that function with complex and indirect links to characteristics of the interventions and outcomes [45]. In contrast, TPEIs create new roles and interactions and can affect the collaborative networks. It can be an event in the history of a system with which it interacts, leading to the evolution of new structures before integration into routine practice [42].

We acknowledge some limitations in this work. The results are sensitive to the country, and other themes could have emerged in another country because practices and values related to health and healthcare are culture-dependent. Participants did not provide feedback on findings. A few HCPs explained that they lacked training, and theoretical frameworks and evidence-based data were often absent from HCPs’ comments. However, they are the ones experiencing the TPE. We lacked the studies to compare our results, but that’s also the originality of the work.

Nevertheless, this study has some strengths. Qualitative data were reported according to COREQ criteria.

Participants were HCPs providing TPE programs for patients with various conditions and in various environments. This maximization sampling variation ensured that a diverse sample of HCPs was recruited, thus supporting credibility and authenticity. Moreover, the coding process resulted from a triangulation including the perspectives of a health psychologist, a sociologist, and a clinician/epidemiologist who provided different insights for a deeper and broader understanding of findings.

### Perspectives

TPEIs may work best if they are tailored to local contexts rather than being completely standardized [43]; however, adaptation to each context should also be identified and analyzed. To understand the mechanisms underlying TPEI effectiveness, one must differentiate what belongs to the intervention itself and what belongs to the context or interaction with the context and how all these entities interact with each other. Thus, describing a list of elements of the context is not sufficient to describe the TPEI, and analyses should also focus on how contextual features might significantly alter the course and impact of an intervention and which elements of the intervention interact and how they interact with elements of the context [41].
